# No Signs of Inflammation during Knee Surgery with Ischemia: A Study Involving Inhaled Nitric Oxide

**DOI:** 10.1155/2014/620281

**Published:** 2014-09-18

**Authors:** Lars Hållström, Claes Frostell, Anders Herrlin, Eva Lindroos, Ingrid Lundberg, Anne Soop

**Affiliations:** ^1^Department of Clinical Science Intervention and Technology, Karolinska University Hospital-Karolinska Institute, 141 86 Stockholm, Sweden; ^2^Department of Physiology and Pharmacology, Anesthesiology and Intensive Care Medicine, Karolinska University Hospital-Karolinska Institute, 141 86 Stockholm, Sweden; ^3^Department of Orthopedic Surgery, Karolinska University Hospital-Karolinska Institute, 171 76 Stockholm, Sweden; ^4^Rheumatology Unit, Department of Medicine, Center for Molecular Medicine, Karolinska University Hospital-Karolinska Institute, 171 76 Stockholm, Sweden

## Abstract

Nitric oxide donors and inhaled nitric oxide (iNO) may decrease ischemia/reperfusion injury as reported in animal and human models. We investigated whether the attenuation of reperfusion injury, seen by others, in patients undergoing knee arthroplasty could be reproduced when patients had spinal anesthesia. 45 consecutive patients were randomized into three groups (*n* = 15). Groups 1 and 3 were receiving iNO 80 ppm or placebo (nitrogen, N_2_) throughout the entire operation, and group 2 only received iNO in the beginning and at the end of the operation. Blood samples were collected before surgery, at the end of the surgery, and 2 hours postoperatively. Muscle biopsies were taken from quadriceps femoris muscle before and after ischemia. There were no increases in plasma levels of soluble adhesion molecules: ICAM, VCAM, P-selectin, E-selectin, or of HMGB1, in any of the groups. There were low numbers of CD68+ macrophages and of endothelial cells expression of ICAM, VCAM, and P-selectin in the muscle analyzed by immunohistochemistry, prior to and after ischemia. No signs of endothelial cell activation or inflammatory response neither systemically nor locally could be detected. The absence of inflammatory response questions this model of ischemia/reperfusion, but may also be related to the choice of anesthetic method EudraCTnr.

## 1. Introduction

Extremity surgery using a tourniquet to provide a bloodless surgical field has been utilized in several human studies as a model of ischemia/reperfusion (I/R) [1–4]. Different anesthetic methods, including general and regional anesthesia [4], have been used in order to influence the oxidative stress and inflammatory response seen in these models, and evaluation of the inflammatory response has been done by measuring inflammatory variables in blood [1–4] and muscle biopsies [2].

The ischemia and reperfusion (I/R) injury have an essential role regarding the pathogenesis in a number of clinical conditions such as stroke, myocardial infarction, and organ transplantation [5–7]. Reperfusion, necessary in providing the postischemic tissue with oxygen and other metabolic substrates, results also in acceleration of the cellular necrotic process and may cause the same consequences as prolonged ischemia [5, 8]. During reperfusion, the formation of reactive oxygen species (ROS) stimulates cytokine and chemokine production through nuclear factor *κ*B (NF-*κ*B) activation. Also, ROS may cause an imbalance between the increased expression of different adhesion molecules on leukocytes and vascular endothelium and the reduced amounts of the antiadhesive nitric oxide (NO) [8–10].

Microvascular barrier disruption with edema and diminished perfusion occurs as a result of the neutrophil adhesion, clot formation, and emigration of neutrophils to the extravascular compartment, a process linked to increased expression of adhesion molecules [8, 11]. NO-donors (e.g., nitroprusside, diethylamine-NO, and S-nitrosoglutathione) have been demonstrated to reduce expression of adhesion molecules (ICAM; intercellular adhesion molecule, VCAM; vascular adhesion molecule, and P-selectin), whereas inhibition of NO-synthesis augmented the expression in human endothelial cells [9, 12, 13]. Therefore, the balance between ROS and NO seems to be an important factor regarding the leukocyte and platelet adhesion induced by I/R. The major site of adhesion (rolling) is the postcapillary venule where the interaction between leukocytes and endothelium occurs, all dependent on adhesion molecules [14].

Inhaled NO (iNO) attenuated the inflammatory response in patients undergoing knee surgery with a bloodless technique using a tourniquet having general anesthesia [15]. The exogenous administration of NO has also been shown to exert anti-inflammatory properties in other I/R settings, both animal [16–18] and human [19].

We aimed to investigate whether knee surgery in regional anesthesia during tourniquet could be used as a model to study I/R with respect to local and systemic inflammatory activation. In addition, we aimed to study the effects of iNO in this context. Therefore we performed a randomized controlled study with the intention to evaluate the potential inflammatory modulatory effect of iNO in this model.

## 2. Materials and Methods

### 2.1. Patients and Study Design

The protocol was approved by the Human Research Ethics Committee at Karolinska Institutet, Stockholm, and the Swedish Medical Product Agency. Written informed consent was obtained from each patient.

**Figure 1:**
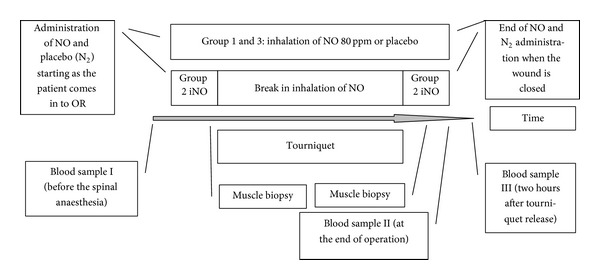
Flow diagram. iNO: inhaled nitric oxide, N_2_: nitrogen (placebo). Groups 1 (iNO) and 3 (placebo) received iNO 80 ppm or placebo (N_2_) throughout the entire operation. Group 2 (piNO) has a break in NO administration during the period of activated tourniquet and administration of NO restarts approximately 2 minutes before the tourniquet release.

Patients (*n* = 45) planned for knee arthroplasty in spinal anesthesia by one specific orthopedic surgeon (AH) at the Karolinska University Hospital, Huddinge, Stockholm, Sweden, were consecutively included in the study (Table 1). The study design is presented in Figure 1. The patients were randomized into three groups (*n* = 15 in each group), and the surgeon, the anesthetic, and the laboratory staff were blinded to the patient. Randomization, using sealed envelopes, occurred when the patients had arrived to the operating theatre. Group 1 received iNO 80 ppm, and group 3 received placebo (N_2_), throughout the entire operation, whereas group 2, partial iNO (piNO), received iNO 80 ppm from the beginning until tourniquet activation (300 mmHg) with a break during ischemia until the blood circulation was restored (release of tourniquet).

An intravenous line, 18 or 20 gauge, was inserted in the cubital fossa or in the forearm for blood sampling (BD Venflon). Spinal anesthesia was performed by an anesthesiologist who is not participating in the study. 0.5% bupivacaine in a range of 12.5–17.5 mg and 0.04% morphine 0.1-0.2 mg were given depending on the individual patient. Thereafter, an oxygen mask, covering mouth and nose, with 5 liter/min oxygen (Eco Oxygen Mask, Intersurgical Ltd., UK) mixed with NO or placebo, was applied. Blood samples for methemoglobin were taken hourly. Surgery and muscle biopsies were performed by the same orthopedic surgeon (AH). The muscle biopsies were taken before tourniquet activation and within 5 minutes after the release.

During surgery the patients were continuously sedated with propofol (0.5–4.0 mg/kg BW/hour).

### 2.2. Laboratory Investigations

Methemoglobin was measured with an ABL800 Flex blood gas instrument (Radiometer Medical, Brønshøj, Denmark). Blood for plasma analysis was collected in EDTA tubes and centrifugated at 4000 rpm 10 minutes in +4°C. The plasma was stored at −80°C for subsequent analysis. Plasma levels of ICAM (intercellular adhesion molecule), VCAM (vascular adhesion molecule), E-selectin, and P-selectin were measured according to manufacturer's instructions using a multiplex ELISA kit (R&D systems, Abingdon, UK), designed for use with a Luminex analyzer. HMGB1 (high mobility group box protein 1) was analyzed using ELISA (Shino-Test Corporation, Japan).

### 2.3. Administration of Inhaled Nitric Oxide and Placebo (Nitrogen, N_2_)

NO (final blended gas at a concentration of 80 ppm NO in inspired gas, INOmax, Ikaria Inc., Hampton, NJ, USA) or placebo (nitrogen, N_2_, Ikaria Inc.) was administered through the INOvent delivery system (Datex-Ohmeda Inc., Madison, USA). Nitrogen dioxide (NO_2_) was constantly measured (INOvent) in inspired air as recommended by the manufacturer.

### 2.4. Muscle Biopsies

Muscle biopsies, measuring approximately 5 × 5 mm, were taken from musculus vastus medialis before and after the tourniquet. The second biopsy was taken from the same area as the first one. The muscle biopsies were immediately frozen in isopentane (C_5_H_12_) with liquid nitrogen and stored at −80°C for subsequent analysis. Biopsies from seven patients in each group were analyzed. These were selected from the patients where the histopathology of the pre-and postischemic biopsy was good.

### 2.5. Immunohistochemistry

The muscle biopsy specimens were analyzed regarding ICAM, VCAM, P-selectin, and CD68/macrophage expression using immunohistochemistry technique. Consecutive 7 *µ*m transverse cryostat sections were mounted on glass slides and air dried for 30 minutes before fixation in cold (+4°C) acetone for 3.5 minutes (30 seconds in 50% acetone and 3 minutes in 100%). The slides were then rinsed in phosphate buffered saline (PBS) for 10 minutes and then treated with 1% H_2_O_2 _+2% NaN_3 _in room temperature (RT) for 60 minutes in darkness to eliminate endogenous peroxidase activity. The slides were then rinsed 3 × 3 minutes in PBS and thereafter incubated with 1% horse serum (Vector Laboratories Inc., Burlingame, CA, USA) in PBS for 15 minutes to avoid nonspecific binding. Primary antibodies (anti-CD54/ICAM-1, mouse IgG1, Serotec, Oxford, UK; anti-CD106/VCAM-1, mouse IgG1, BD Pharmingen, San Diego, CA, USA; anti-CD62P/P-selectin, mouse IgG1, Santa Cruz Biotechnology Inc., Heidelberg, Germany; anti-CD68/macrophages, mouse IgG1, Dako A/S, Glostrup, Denmark) were applied overnight to the sections at RT in a humid chamber. As a negative control we used an irrelevant mouse antibody (isotype-matched irrelevant, mouse IgG1, Dako A/S, Glostrup, Denmark) as primary antibody.

After washing in PBS 3 × 3 minutes secondary antibodies (biotinylated, horse anti-mouse IgG, Vector Laboratories Inc., Burlingame, CA, USA) diluted in PBS and 1% normal horse serum were applied to the sections for 30 minutes at RT followed by washing in PBS for 3 × 3 minutes. The sections were then incubated with avidin-biotin complex (avidin-biotin-horseradish peroxidase complex, Vectastain, ABC-HP kit, Vector Laboratories Inc., Burlingame, CA, USA) for 45 minutes in RT. After washing in PBS for 3 × 3 minutes peroxidase reactions were developed using 3,3′-diaminobenzidine (DAB substrate kit for peroxidase, Vector Laboratories Inc., Burlingame, CA, USA) for 7 minutes followed by 3 × 3 minutes washing in PBS. The sections were then lightly counterstained with Mayer's hematoxylin and mounted using buffered glycerol.

### 2.6. Evaluation of Staining

Tissue sections were analyzed using Reichert-Jung Polyvar 2 (Leica Microsystems, Wetzlar, Germany). Semiquantification (scoring 0, 1+, 2+, and 3+) was performed by two independent investigators, blinded with the respect to the study groups. The number of cells positively stained for ICAM, VCAM, P-selectin, and CD68/macrophages was estimated from the whole tissue sections.

### 2.7. Statistics

Statistical analysis to evaluate the effect of treatment and the effect of I/R over time was performed using two-factor repeated-measures analysis of variance (ANOVA) with post hoc comparisons (Scheffè) when applicable, whereas sign test was used for the nonparametric data (muscle biopsies). Statistica 5.5 for Windows System was used for analysis. 15 individuals per sample group will, with a power of 90%, enable noticing a difference of 15% of soluble plasma P-selectin with a significance level of 5% (alpha = *P* value).

## 3. Results

**Figure 2:**
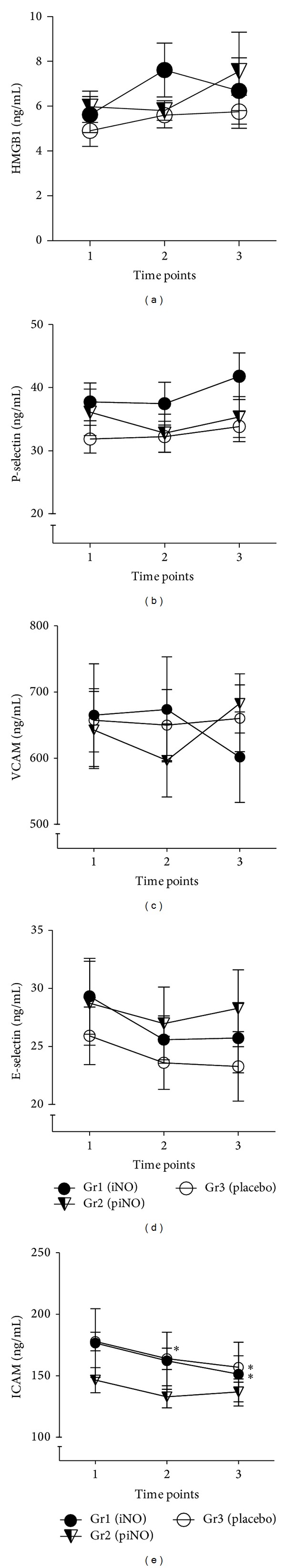
Effects on soluble adhesion molecules and HMGB1 in plasma. A significant decrease in ICAM was observed in group 1 (iNO) at time point 3 (two hours after tourniquet release) and in group 3 (placebo) at time points 2 (at the end of operation) and 3. No differences were observed between the groups. *P < 0.05 compared with baseline. Mean ± SEM.

Plasma levels of HMGB1, P-selectin, VCAM, and E-selectin all remained unaltered with no differences between groups at any time point. Moreover, there were no significant changes within the three groups. However, soluble ICAM showed a small, but significant, decrease in two of the three groups (Figure 2(e)).

**Figure 3:**
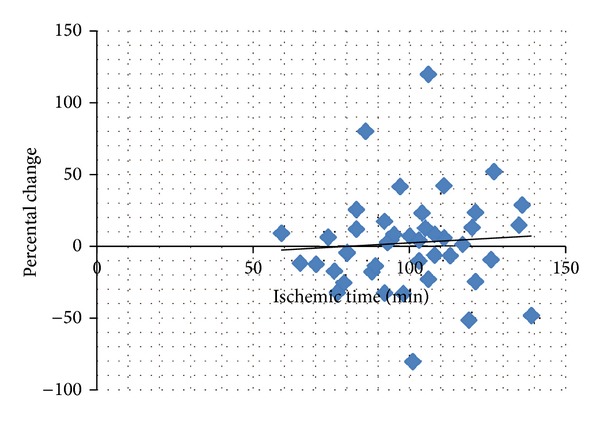
Correlation between percental change (blood sample at time points one and two) and ischemic time, exemplified by soluble P-selectin (all three groups). *R* ^2^ = 0.0048, ns.

No correlation could be demonstrated between plasma levels of soluble adhesion molecules and HMGB1 and ischemic time (E-selectin *R* ^2^ = 0.0002, ICAM *R* ^2^ = 0.0006, VCAM *R* ^2^ = 0.00002, and HMGB1 *R* ^2^ = 0.0858). As an illustration the lack of correlation for soluble P-selectin is shown in Figure 3.

In the repeated muscle biopsies we found scattered CD68+ macrophages, but there were no clusters of inflammatory cells. There was no difference in the expression of CD68+ macrophages in the biopsies taken after the ischemia period. The expression of adhesion molecules was consistently found in endothelial cells; ICAM was equably scattered between muscle cells while VCAM and P-selectin were found mainly in larger vessels (arterioles and venules). There were no differences in number of positive capillaries or cells observed between the three groups before and after ischemia and there were no within group changes concerning number of expressed adhesion molecules after the ischemia compared to before.

**Figure 4:**
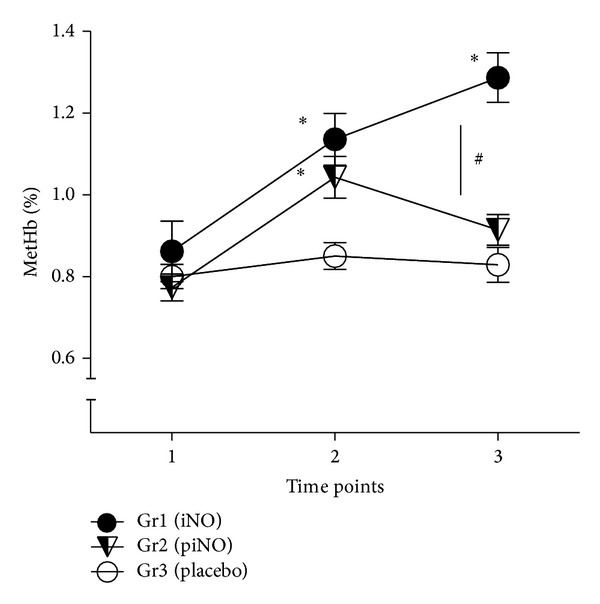
There was a significant increase in methemoglobin levels compared with baseline for the iNO treated patients and a significant difference over time in contrast to the placebo treated (*P < 0.05); also, a significant difference between the two iNO groups was observed (^#^ *P* < 0.05). Groups 1 (iNO) and 3 (placebo, N_2_) received iNO 80 ppm or placebo throughout the entire operation, whereas group 2 (piNO) only received iNO 80 ppm in the beginning and at the end of the operation. Time point 1; before the spinal anesthesia, time point 2; before tourniquet activation, and time point 3; after tourniquet release. Mean ± SEM.

Methemoglobin increased significantly over time in the iNO treated groups compared to the placebo treated, thus, serving as a marker of exposure to iNO [20] (Figure 4).

## 4. Discussion

In this study we found no support in the peripheral circulation for endothelial cell activation measured by soluble adhesion molecules or for an inflammatory response measured by HMGB1 levels within 2 hours after ischemia in patients undergoing knee surgery using spinal anesthesia. A small decrease in soluble ICAM was observed in two of the three study groups. Furthermore, there were no signs of endothelial cell activation or of an increased number of macrophages homing into the muscle after the ischemia period in the thigh muscle.

To our surprise, no signs of endothelial cell activation or an inflammatory response could be observed neither in plasma nor in muscle biopsies in this clinical human study with reperfusion following an ischemic event. As a consequence, influence neither was seen nor could be expected, regarding the randomized iNO exposure on the measured parameters.

In a recent study iNO has been demonstrated to provide an improvement in penumbral blood flow and neuronal survival in stroke or other ischemic conditions [21].

In contrast to the results in the present study, Mathru et al. found, in anaesthetized patients going through knee surgery, significantly increased inflammation (augmented expression of soluble P-selectin, CD11b/CD18 on neutrophils, plasma lipid hydroperoxide levels, activated nuclear factor-*κ*B, and myeloperoxidase activity in muscle biopsies as well as content of conjugated dienes); also, iNO significantly attenuated this inflammatory response [15]. However, there are some dissimilarities between the work of Mathru et al. and the current study. Firstly, in the present study all the patients had spinal anesthesia in concordance to the clinical routine, in contrast to Mathru et al. who used general anesthesia with thiopental and isoflurane. The patients in our study were thus conscious although they were modestly sedated. The choice of anesthetic method may have importance, since different anesthetic drugs have been reported to have influence on inflammation in various settings. For example, in an ovine endotoxin model, isoflurane anesthesia compared to consciousness resulted in significantly impaired renal function in combination with enhanced neutrophil activity and accumulation in the kidney [22]. Interestingly, in anaesthetized rats, isoflurane protected the hepatic tissue compared to pentobarbital sodium in an I/R model [23], whereas isoflurane did not attenuate the liver damage yet ketamine did in an endotoxin model [24]. Also, the anesthetic drug propofol has, in the I/R setting, been proposed to possess antioxidant capabilities, both in rats [25] and in humans [2, 4, 26, 27]. However, lack of beneficial effects against myocardial I/R injury (rat) using propofol has also been reported [28]. Further, in patients undergoing coronary artery bypass graft surgery having remote ischemic precondition, propofol anesthesia could not, compared to isoflurane, decrease myocardial damage [29]. Also, local anesthetics have been shown to possess various effects on polymorphonuclear leukocytes such as inhibiting migration, enzyme release, and superoxide anion generation. This impairment of neutrophil function may contribute to an anti-inflammatory and immunosuppressive effect [30]. The use of epidural anesthesia combined with general anesthesia for surgery has been suggested as a safe and reliable method in reducing overall postoperative complication rate and the incidence of cardiovascular failure and major infections complications [31]. Park et al. found recently, when comparing arterial lactate levels in patients undergoing total knee arthroplasty having sevoflurane or spinal anesthesia, a higher lactate increase during sevoflurane anesthesia, thus suggesting a lower production of ischemic metabolites in the spinal anesthesia group [32]. Improved microcirculation during organ inflammation has been indicated in an experimental rat model using epidural anesthesia [33]. A positive effect on the tissue perfusion by neuraxial block may modulate the inflammatory response during ischemia/reperfusion.

Hence, there may be some inconsistencies regarding the influence of different anesthetic methods in various inflammatory settings, and since we used propofol sedation in addition to the spinal anesthesia it could be speculated that this compound may have attenuated the inflammatory response in the ischemic tissue. Secondly, our patients were almost twice as old compared to patients in the Mathru et al. study [15]. In humans, a positive correlation between age and plasma concentration of soluble ICAM and VCAM has been observed [34]. Therefore, it seems unlikely that the higher age in the present study could explain the difference in the inflammatory response. Soluble adhesion molecules have been reported to increase during aging (rats); also, when treated with LPS older animals showed a more pronounced expression of these markers [35].

Total tourniquet duration in the present study was 60–140 minutes which is in concordance with the approximately two hours presented by Mathru et al. [15].

The patients in the current study were all having the same type of surgery performed by the one surgeon: knee arthroplasty. This design was established in order to optimize the reliability and validity of the study results.

There is not always an augmentation of the adhesion molecule expression in the I/R injury. In a major human I/R setting, liver transplantation, no increase in these parameters were seen [19]. Conversely in other species, it has been shown differently. In a canine model of hepatic I/R, the expression of ICAM, VCAM, P-selectin, and E-selectin was increased after 60 minutes of warm ischemia [36]. Further, P-selectin synthesis is increased by stimulation of TNF-*α* or LPS in murine but not in human endothelial cells [37]. HMGB-1 is considered a late mediator of sepsis in animal models [38, 39] and has been correlated with outcome (survivors/non-survivors) in septic patients [38]. However, in trauma patients a peak was seen as early as 2–6 hours after hospital admittance [40]. Nevertheless, it has been shown that HMGB-1 is of vital importance in mediating injury in settings of I/R; for example, in mice HMGB1 protein expression in the liver was upregulated one hour after reperfusion and after 30 minutes in the heart [41, 42]. In patient studies with cerebral and myocardial ischemia, respectively, elevated levels of HMGB1 were seen within 24 hours [43, 44]. The values of HMGB1 in the present study are in the expected normal range [45, 46]. Hence, species difference, time in relation to I/R event, patient comorbidity, and cardiovascular disease risk factors [47] may be variables influencing these parameters.

A decrease in soluble ICAM was observed over time in the placebo group and in the group receiving iNO throughout surgery, a finding corresponding with an earlier study by Huda et al., where a decrease in soluble ICAM was seen after tourniquet in patients undergoing knee surgery. Notably this was seen in conjunction with an increase in ICAM mRNA in muscle biopsies [1]. In our study the ICAM staining in the muscle biopsies did not confirm this decrease in soluble ICAM. Consequently, we do not interpret these small changes as clinically relevant. Soluble forms of adhesion molecules are detectable in biological fluids and their concentrations may reflect an increased expression on the cellular level due to an inflammatory response [47, 48]. However, the measurement of mRNA has been questioned and demonstrated to have rather poor correlation with corresponding protein profiles [49].

In this study a maximal dose of iNO was administrated in order to evaluate the hypothesized anti-inflammatory effect, throughout the whole period of surgery or with a break when the tourniquet was activated. This design was used in order to investigate if iNO administered when there was circulation to the leg could modulate the inflammatory response, hypothesizing that the administration of iNO may create a repository of NO [50, 51]. Expression of inflammation in this present study was evaluated using two standard methods: ELISA and immunohistochemical techniques.

Although no beneficial effect of iNO administration in the present study could be observed, we have on the other hand no traces of harm neither locally nor systemically. However, the study was not designed for a safety statement regarding iNO.

## 5. Conclusions

In this I/R setting in patients undergoing knee arthroplasty with a tourniquet time range 60 to 140 minutes in spinal anesthesia, no increase in the inflammatory response could be observed systemically or in the local muscle tissue. Hence, no statement of putative influence of iNO could be formulated. Further work could be addressed to the use of different I/R models and other types of NO donors as well as anesthetic methods.

## Figures and Tables

**Table 1:** Patient characteristics.

	Group 1 (iNO)	Group 2 (piNO)	Group 3 (placebo)
Patients, *n*	15	15	15
Gender (F/M)	8♀/7♂	10♀/5♂	13♀/2♂
Mean age, years	63 ± 14	65 ± 9	64 ± 9
Mean BMI	27.3 ± 4.6	29.8 ± 6.1	30.8 ± 5.4
Tourniquet time, min	101 ± 20 range 74–127	103 ± 19 range 70–140	95 ± 19 range 60–121
Total duration of surgery, min	170 ± 31 range 114–223	168 ± 20 range 125–207	156 ± 32 range 98–204
Total time of iNO, min	196 ± 36 range 131–259	97 ± 16 range 71–132	—

iNO: inhaled nitric oxide, piNO: partial iNO. Groups 1 (iNO) and 3 (placebo, N_2_) received iNO 80 ppm or placebo throughout the entire operation, whereas group 2 (piNO) received iNO 80 ppm in the beginning and at the end of the operation, with a break in iNO administration during the period of activated tourniquet. Values are expressed as mean ± SD if not stated otherwise.
